# Incidence of bacterial and fungal infections in Polish pediatric patients with acute lymphoblastic leukemia during the pandemic

**DOI:** 10.1038/s41598-023-50093-5

**Published:** 2023-12-18

**Authors:** Joanna Zawitkowska, Katarzyna Drabko, Monika Lejman, Adrian Kowalczyk, Krzysztof Czyżewski, Magdalena Dziedzic, Kamila Jaremek, Patrycja Zalas-Więcek, Anna Szmydki-Baran, Łukasz Hutnik, Wojciech Czogała, Walentyna Balwierz, Iwona Żak, Małgorzata Salamonowicz-Bodzioch, Bernarda Kazanowska, Grażyna Wróbel, Jowita Frączkiewicz, Krzysztof Kałwak, Renata Tomaszewska, Tomasz Szczepański, Olga Zając-Spychała, Jacek Wachowiak, Marcin Płonowski, Maryna Krawczuk-Rybak, Aleksandra Królak, Tomasz Ociepa, Tomasz Urasiński, Filip Pierlejewski, Wojciech Młynarski, Justyna Urbańska-Rakus, Katarzyna Machnik, Sonia Pająk, Wanda Badowska, Tomasz Brzeski, Katarzyna Mycko, Hanna Mańko-Glińska, Agnieszka Urbanek-Dądela, Grażyna Karolczyk, Agnieszka Mizia-Malarz, Weronika Stolpa, Katarzyna Skowron-Kandzia, Jakub Musiał, Radosław Chaber, Ninela Irga-Jaworska, Ewa Bień, Jan Styczyński

**Affiliations:** 1 Department of Pediatric Hematology, Oncology and Transplantology, Medical University of Lublin, Lublin, Poland; 2 Independent Laboratory of Genetic Diagnostics, Medical University of Lublin, Lublin, Poland; 3 Student Scientific Society of Department of Pediatric Hematology, Oncology and Transplantology, Medical University of Lublin, Lublin, Poland; 4 Department of Pediatric Hematology and Oncology, Collegium Medicum in Bydgoszcz, Nicolaus Copernicus University in Torun, Torun, Poland; 5 Department of Microbiology, Collegium Medicum in Bydgoszcz, Nicolaus Copernicus University in Torun, Torun, Poland; 6 Department of Hematology and Pediatrics, Medical University of Warsaw, Warsaw, Poland; 7 Department of Pediatric Oncology and Hematology, Intitute of Pediatrics, Jagiellonian University Medical College, Kraków, Poland; 8 Department of Microbiology, University Children’s Hospital, Jagiellonian University Medical College, Kraków, Poland; 9 Department of Pediatric Bone Marrow Transplantation, Oncology and Haematology, Wroclaw Medical University, Wroclaw, Poland; 10 Department of Pediatrics, Hematology and Oncology, Medical University of Silesia, Katowice, Poland; 11 Department of Pediatric Oncology, Hematology and Transplantology, Poznan University of Medical Sciences, Poznan, Poland; 12 Department of Pediatric Oncology, Hematology, Medical University of Bialystok, Bialystok, Poland; 13 Department of Pediatrics, Hemato-Oncology and Gastroenterology, Pomeranian Medical University, Szczecin, Poland; 14 Department of Pediatrics, Oncology and Hematology, Medical University of Lodz, Lodz, Poland; 15 Unit of Pediatric Hematology and Oncology, City Hospital, Chorzow, Poland; 16 Collegium Medicum University of Warmia and Mazury in Olsztyn, Olsztyn, Poland; 17 Department of Pediatric Oncology and Hematology, Collegium Medium of Jan Kochanowski University in Kielce, Kielce, Poland; 18 Department of Oncology, Hematology and Chemotherapy, Upper Silesia Children’s Care Health, Medical University of Silesia, Katowice, Poland; 19 Clinic of Pediatric Oncology and Hematology, Faculty of Medicine, University of Rzeszow, Rzeszow, Poland; 20 Department of Pediatrics, Hematology, Oncology and Endocrinology, Medical University of Gdansk, Gdańsk, Poland

**Keywords:** Microbiology, Oncology

## Abstract

The most common complications related to the treatment of childhood acute lymphoblastic leukemia (ALL) are infections. The aim of the study was to analyze the incidence and mortality rates among pediatric patients with ALL who were treated in 17 Polish pediatric hematology centers in 2020–2021 during the pandemic. Additionally, we compared these results with those of our previous study, which we conducted in the years 2012–2017. The retrospective analysis included 460 patients aged 1–18 years with newly diagnosed ALL. In our study, 361/460 (78.5%) children were reported to have microbiologically documented bacterial infections during chemotherapy. Ten patients (2.8%) died due to sepsis. Fungal infections were reported in 99 children (21.5%), of whom five (5.1%) died due to the infection. We especially observed an increase in bacterial infections during the pandemic period compared to the previous study. The directions of our actions should be to consider antibiotic prophylaxis, shorten the duration of hospitalization, and educate parents and medical staff about complications (mainly infections) during anticancer therapy. It is necessary to continue clinical studies evaluating infection prophylaxis to improve outcomes in childhood ALL patients.

## Introduction

The development of new diagnostic methods and the use of effective therapeutic protocols improved the outcomes of childhood acute lymphoblastic leukemia (ALL) patients. Currently, the long-term survival rate in children with ALL exceeds 90%^1,2^. However, the complications related to ALL treatment remain an unsolved challenge, especially bacterial and fungal infections. Literature reports show that infection-related mortality (IRM) in patients with ALL accounts for 30% of deaths and 64% of treatment-related mortality. Most deaths are due to bacterial infections (68%), followed by fungal and viral infections (20% and 12%, respectively)^3^. Risk factors for the development of infections in patients undergoing anticancer therapy include Caucasian race, female sex, age (higher risk in infants and adolescents), Down syndrome, steroid use, the presence of a central venous catheter, and a prolonged neutropenic phase^2,4,5^. The need to reduce the dose of cytostatic drugs or even stop anticancer treatment due to infection may significantly increase the risk of relapse^6–8^. It is very important to be aware of the types of pathogens that cause infection in children and adolescents with ALL to establish infection prophylaxis strategies, including bacterial and fungal infection prevention.

In this study, the incidence of bacterial and fungal infections, type of pathogens and mortality rate among pediatric ALL patients in Polish hemato-oncology centers were recorded during the pandemic (2020–2021). Additionally, we compared these results with those of our previous study, which we conducted in the years 2012–2017. Data on viral infections for 2020–2021 were published elsewhere^8^.

## Results

### Bacterial infections

**Figure 1:**
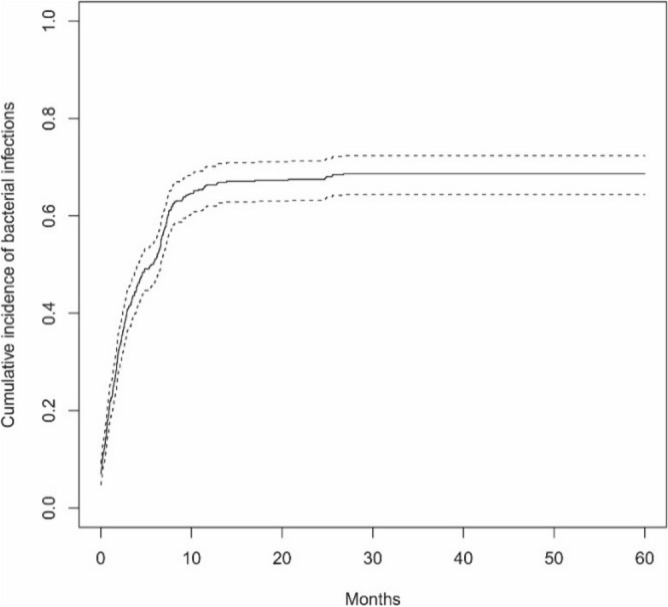
Cumulative incidence of bacterial infections from the time of ALL diagnosis (dotted lines indicate 95% confidence interval).

**Figure 2:**
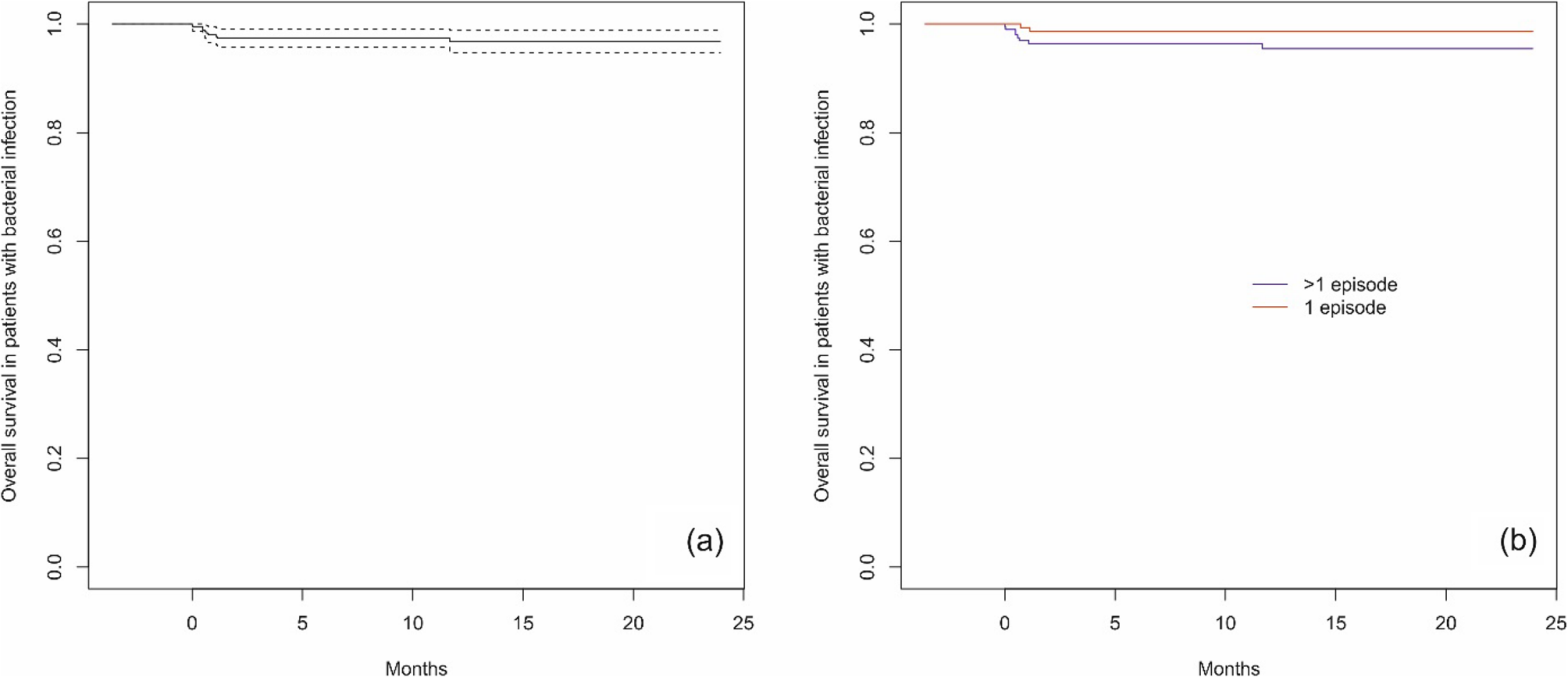
Overall survival in patients with bacterial infections and ALL diagnosis (**a**) and by subgroups (**b**) (dotted lines indicate 95% confidence interval).

Bacterial infections were reported in 361/460 (78.5%) patients; 158 (43.8%) patients had 1 bacterial infection, and 203 (56.2%) patients had more than 1 bacterial infection. In the group of patients with more than 1 infection, a total of 715 infections were reported. There were no significant differences between patients with 1 infection and those with more than 1 infection depending on sex (p = 0.984) or age at ALL diagnosis (p = 0.363). Ten patients (2.8%) died due to bacterial infection, including 2 patients with 1 infection and 8 patients with more than 1 infection. The median time from the time of ALL diagnosis to the beginning of an infection was 141.5 days, while the median infection duration was 10 days, and there were no significant differences between the analyzed groups (Table 1). The bloodstream was the most common site of bacterial infection (n = 289; 80%), followed by the gastrointestinal tract (n = 193; 53.5%) and the urinary tract (n = 161; 44.6%). Overall, 143/289 (49%) gram-positive and 146/289 (51%) gram-negative pathogens were detected in the blood. The type of isolates in patients with bloodstream infections are shown in Table 2. Sepsis was the cause of death in 10 children (2.8%). The median survival time for ALL patients with bacterial infections was 5.64 months [95 CI 4.10; 6.62]. At 6 months from the time of ALL diagnosis, the cumulative incidence of bacterial infections was 51.1% [95 CI 46.6; 55.2], and at 12 months, it was 66.3% [95 CI 62.0; 70.2] (Fig. 1). Overall survival (OS) in patients with bacterial infections was 96.7% [95 CI 94.7; 98.9] (Fig. 2a). There were no significant differences in OS between patients with 1 and > 1 bacterial infection (p = 0.100) (Fig. 2b).

**Table 1:** Groups characteristics for bacterial infections (patients with 1 and > 1 infections).

Characteristics	Total group	1 infection	> 1 infections	p
Patients				
Number of patients	361	158	203	
Sex, n (%)				
Female	170 (47.1)	75 (47.5)	95 (46.8)	0.984
Male	191 (52.9)	83 (52.5)	108 (53.2)	
Age at ALL diagnosis, years				
Median (Q1; Q3)	4.81 (2.67;9.21)	5.12 (2.97;8.61)	4.78 (2.54;9.33)	0.363
Death related to infection, n (%)	10 (2.8)	2 (1.3)	8 (3.9)	0.196
Infections				
Number of infections	873	158	715	
Time from ALL diagnosis to infection start, days				
Median (Q1; Q3)	141.50 (58.00;221.25)	135.00 (44.50;218.50)	144.00 (63.00;222.00)	0.933
Infection duration, days				
Median (Q1; Q3)^a^	10.00 (7.00;14.00)	10.00 (7.00;13.25)	10.00 (7.00;14.00)	0.880

*Analysis with chi-square test (sex), Fisher exact test (death) and with Mann–Whitney’s U test for remaining parameters. *ALL* acute lymphoblastic leukemia. ^a^Infection duration defined as time from diagnosis of infection till end of infection/death. Median calculated in a classical way (middle value).

**Table 2:** The type of isolates in bloodstream infections.

The type of infection	Number of bacterial infection episodes (n = 318)	Number of deaths	Time from infection start to death, days
Gram-positive organism	143 (49%)		
*Staphylococci species*	104	2 (0.7%)	19–20
*S. aureus*	12	1 (0.3%)	12
*MR-CNS*	13	–	
*Streptococci species*	11	1 (0.3%)	18
*Enterococcus species*	24	0	
*Bacillus species*	3	–	
*Listeria monocytogenes*	1		
Gram-negative organism	146 (51%)	–	
*Escherichia coli*	51	2 (0.7%)	10–15
*Klebsiella species*	30	2 (0.7%)	6–12
*Pseudomonas species*			
*P. aeruginosa*	6	–	
*Acinetobacter baumani*	3	–	
*Stenotrophomonas maltophilia*	2	–	
*Enterobacter species*	24	1 (0.3%)	18
*Serratia marcescens*	1	–	
Others	29	1 (0.3%)	9

*MR-CNS* methicillin-resistant coagulase-negative Staphylococci.

### Fungal infection

**Figure 3:**
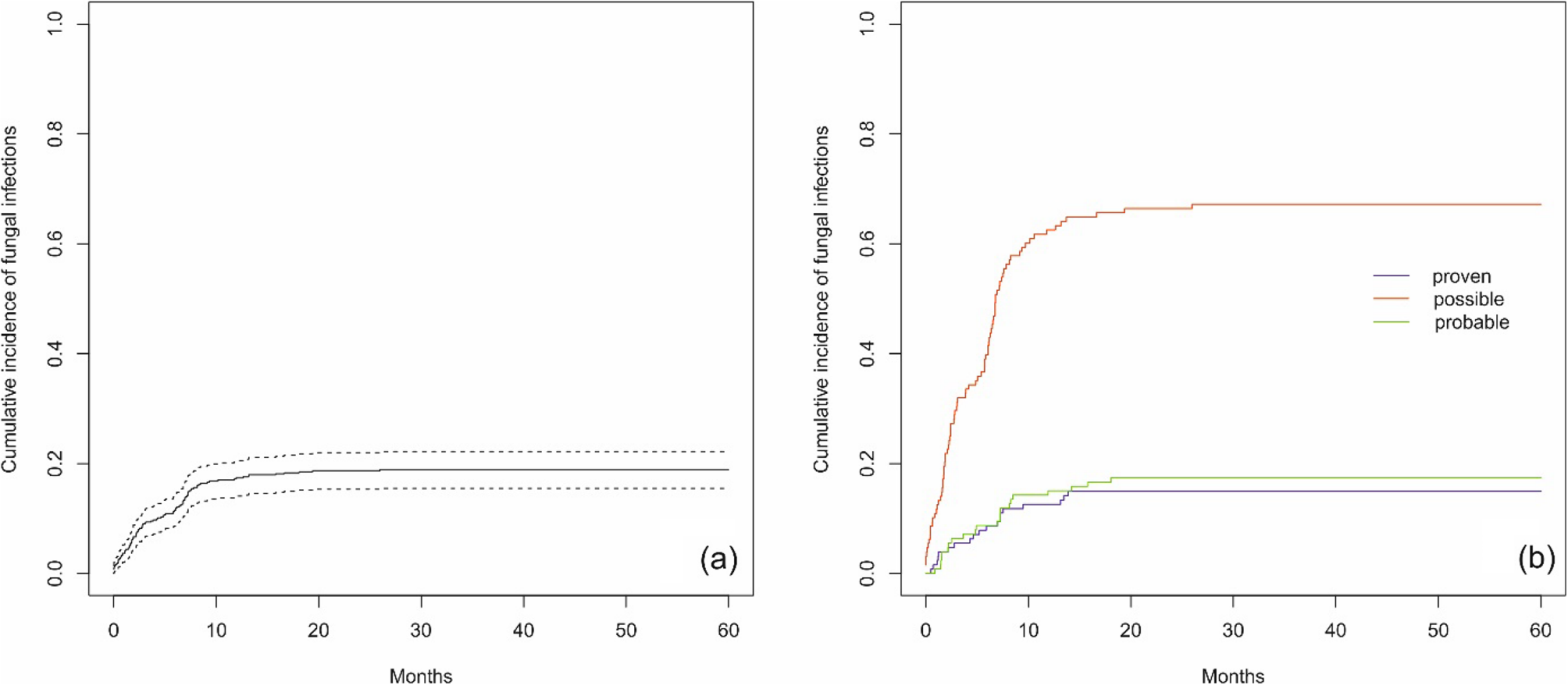
Cumulative incidence of fungal infections from the time of ALL diagnosis (**a**) and by possible, probable and proven fungal infections (**b**) (dotted lines indicate 95% confidence interval).

**Figure 4:**
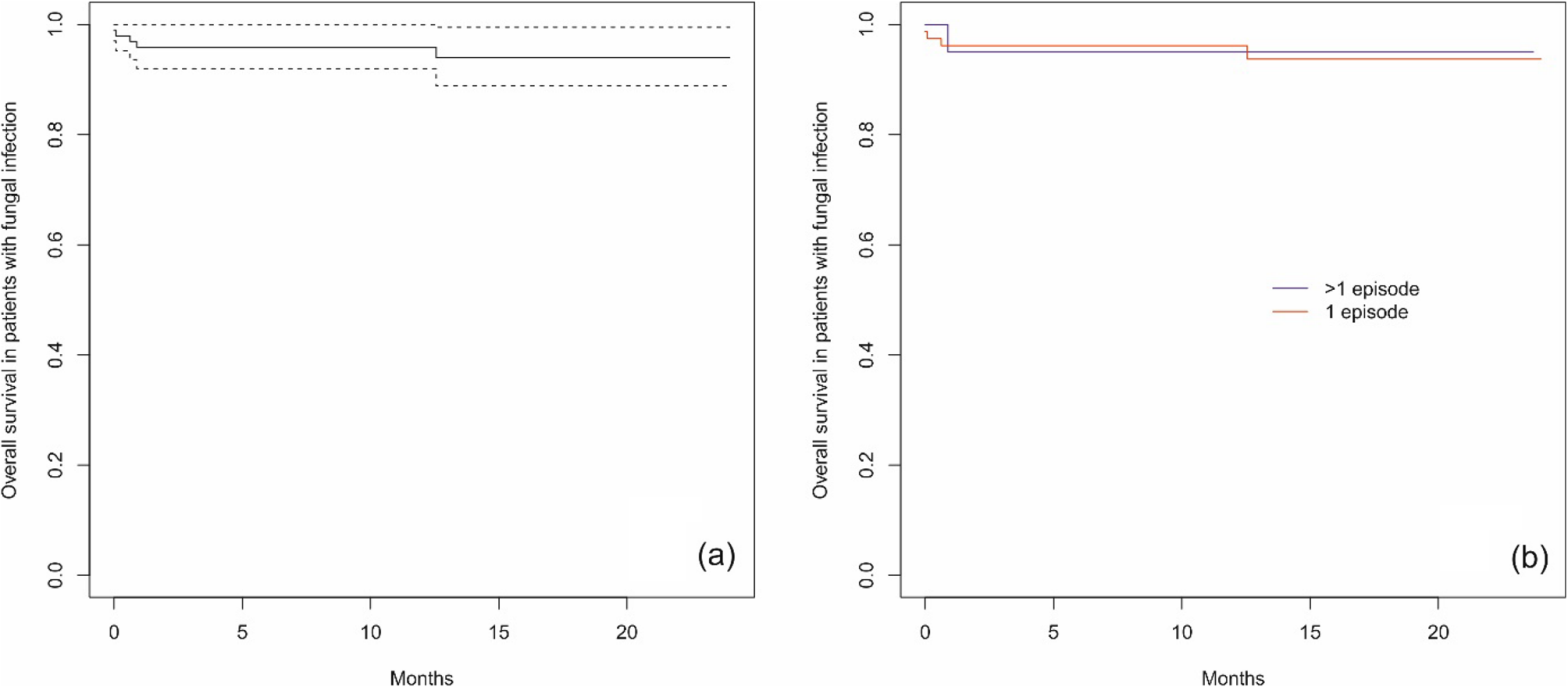
Overall survival in patients with fungal infections and ALL diagnosis (**a**) and by subgroups (**b**) (dotted lines indicate 95% confidence interval).

A total of 99 patients had fungal infections, including 79 (79.8%) patients with 1 infection and 20 (20.2%) patients with more than 1 infection. In the group with more than 1 infection, a total of 51 infections were reported. There was no significant difference between the 2 groups depending on sex (p = 0.549) or age at the time of ALL diagnosis (p = 0.133). Five patients (5.1%) died due to a fungal infection, including 4 patients with 1 infection and one patient with more than 1 fungal infection (2 infections). No significant differences between the analyzed groups were confirmed for the time between ALL diagnosis and the beginning of an infection (p = 0.172) or infection duration (p = 0.056) (Table 3). The type of pathogens (proven infections) is presented in Table 4. Survival curves for the cumulative incidence of fungal infections in ALL patients based on the Kaplan‒Meier survival analysis method were not achieved. The cumulative incidence of fungal infections was 11.5% [95 CI 8.7; 14.2] 6 months after ALL diagnosis, and it was 17.4% [95 CI 14.1; 20.6] 12 months after diagnosis (Fig. 3a). The cumulative incidence of possible fungal infections was 67.7% (probable: 17.7% and proven: 14.6%), and the differences were statistically significant (p < 0.001) (Fig. 3b). OS in patients with fungal infections was 94.0% [95 CI 88.8; 99.5] (Fig. 4a). There were no statistically significant differences in OS between patients with 1 and > 1 fungal infection (p > 0.999) (Fig. 4b).

**Table 3:** Groups characteristics for fungal infections (patients with 1 and > 1 infections).

Characteristics	Total group	1 infection	> 1 infections	p
Patients				
Number of patients	99	79	20	
Sex, n (%)				
Female	43 (43.4)	36 (45.6)	7 (35.0)	0.549
Male	56 (56.6)	43 (54.4)	13 (65.0)	
Age at ALL diagnosis, years				
Median (Q1; Q3)	6.69 (3.72;10.97)	6.87 (4.06;11.05)	4.23 (2.12;9.65)	0.133
Death related to infection, n (%)	5 (5.1)	4 (5.1)	1 (5.0)	> 0.999
Infections				
Number of infections	130	79	51	
Time from ALL diagnosis to infection start, days				
Median (Q1; Q3)	149.00 (52.00;222.00	176.00 (58.00;226.00)	123.50 (37.25;216.75)	0.172
Infection duration, days				
Median (Q1; Q3)^a^	19.00 (10.00;35.00)	20.50 (11.75;44.50)	14.00 (8.00;29.50)	0.056

Analysis with chi-square test (sex), Fisher exact test (death) and with Mann–Whitney’s U test for remaining parameters. *ALL* acute lymphoblastic leukemia. ^a^Infection duration defined as time from diagnosis of infection till end of infection/death. Median calculated in a classical way (middle value).

**Table 4:** Pathogens of proven fungal infection episodes.

The type of pathogen	Number of fungal infection episodes (n = 19)	Number of deaths	Time from infection start to death, days	
Candida species				
* C. glabrata*		2	1 (5.3%)	10
* C. crusei*		2	–	
* C. albicans*		2	1 (5.3%)	19
* C. parapsilosis*		2	–	
* C. guilliermondii*		1	1 (5.3%)	10
* Aspergillus fumigatus*		4	–	
Mucor		3	–	
*Pneumocystis jirovecii*		3	–

### Comparison of the studies during 2012–2017 and 2020–2021

There was a significant increase between the incidence of bacterial infections in the years 2012–2017 (726(/1363, 53.2%) and 2020–2021 (361/460, 78.5%) (OR 0.3; p < 0.0001). No significant differences between the analyzed studies were observed due to sex (OR 1.1; p = 0.3). There was a significant decrease between infection episodes with gram-positive isolates (OR  1.6; p = 0.0004) and no significant differences for infection episodes with gram-positive isolates (OR 0.7; p = 0.09). We did not observe significant differences between patients who survived and those who died (OR 0.9; p = 0.9)^9^.

There was no significant difference between the incidence of fungal infections in the years 2012–2017 (278/1363, 20.4%) and 2020–2021 (99/460, 21.5%) (OR 0.9; p < 0.6). No significant differences between the analyzed studies were observed due to sex (OR 0.7; p = 0.3). When comparing episodes of infections in the years 2012–2017 and 2020–2021 at the proven (respectively 28/406, 6.9% and 19/99, 19.2%), probable (41/406, 10.1% and 23/99, 23.2%) and possible (217/406, 19.2% and 60/99, 60.6%) levels for these studies, statistically significant differences were found (p = 0.001). We did not observe significant differences between patient who survived and those who died (OR 0.5; p = 0.3)^9^.

## Discussion

Data from the literature show that infection-related mortality (IRM) is the main factor influencing the treatment outcomes of pediatric ALL patients. Steroid courses and profound neutropenia were found to be significant risk factors predisposing patients to infectious complications^3^.

In our previous study, 53.2% of the children were reported to have microbiologically documented bacterial infections during chemotherapy, mainly bloodstream infections (71.3%). Twenty children (2.75%) died due to sepsis, and gram-negative bacteria were most frequently implicated in infection-related mortality (IRM). Fungal infections were observed in 20.4% of children with ALL, and 2.9% died due to the infection. Due to the pandemic, we decided to analyze the period 2020–2021 and assess the profile of the incidence of bacterial and fungal infections among pediatric patients with ALL in the same Polish centers. There is a lack of studies investigating the infection profile during the pandemic period. In this study, we observed a significantly increased incidence of microbiologically documented bacterial infections (78.5%) during 2020–2021 compared to 2012–2017. Similarly, the most common source of an infection was the bloodstream (71.3%). The number of infections due to gram-positive isolates was significantly increased in the previous study. The mortality rate was similar. Data related to the incidence of fungal infections, despite new antifungal agents, were comparable to a previous report (21.5% and 20.4%, respectively). The death rate in the study group of patients with fungal infections increased (2.9% in the period 2012–2017 and 5.1% in the period 2020–2021), but the difference was not statistically significant^9^.

The higher frequency of bacterial infection during the years 2020–2021 could have been caused by the COVID-19 pandemic. According to the analyses presented in the 2021 Report "The impact of the COVID-19 pandemic on the oncological care system" developed by the National Research Institute in Poland, when patients were admitted to oncological facilities with suspected cancer, they were quickly diagnosed and received treatment in a timely manner. According to experts, problems appeared at an earlier stage of disease. The authors of the Report indicated that the most important reasons for this state of affairs were patients' fear of visiting hospitals and restrictions on the functioning of primary health care facilities during this period. This seems to be caused by intensive measures to prevent the rapid spread of the epidemic (e.g., the "Stay at Home" campaign), local and global chaos related to our understanding of the dynamics of the virus's spread, or people's greater fear of SARS-CoV-2 infection in the earlier phrases than in the later phases. Additionally, excessive use of teleconsultations contributed to delays in diagnosis^10^. We observed that pediatric patients with advanced disease complicated by infection often visited hemato-oncology centers due to less frequent primary care visits and their families' reluctance to expose them to SARS-CoV-2, which delayed the cancer diagnosis. The second reason is that cancer patients with fever during treatment stayed at home for too long because their parents were afraid of hospitalization.

Filho et al. reported a retrospective study that included patients hospitalized due to infection in a pediatric oncology unit from 2018 to 2021. A total of 168 episodes of infections were identified in 96 patients, resulting in 157 hospitalizations. Among the patients with infections, 62.4% had hematological malignancies, and out of these patients, 74.6% specifically had ALL. *Escherichia coli* (31.9%) was the most prevalent microorganism isolated from the samples. The authors observed no significant change in the number of hospitalizations in the investigated pediatric unit. This stability may be attributed to the specialized care required for cancer patients, the awareness of the children’s families regarding the importance of hospital care, and the institution’s commitment to providing care for patients even amidst the challenges posed by the pandemic^11^.

A multicenter, international, collaborative cohort study assessed the impact of the COVID-19 pandemic on pediatric cancer patients in low- and middle-income countries (LMICs) and high-income countries. The results showed that health care delays and disruptions were particularly common for pediatric cancer patients in LMICs. Staff shortages were reported primarily in LMICs, causing supply chain disruptions, increased chemotherapy drug prices and a lack of personal protective equipment. Overall, these results indicate that the delivery of COVID-19 care occurred in health care systems around the world. Before the pandemic, many health systems in LMICs were already burdened by limited resources and access to care for children with cancer. During the COVID-19 pandemic, these institutions experienced greater staffing and supply shortages, treatment modifications, and a lack of supportive care^12^.

O’Connor et al. reported a large study of the United Kingdom Childhood Acute Lymphoblastic Leukemia Randomized Trial 2003 (UKALL 2003), which included 3126 eligible patients. A total of 249 deaths were recorded, of which 132 (53.0%) were disease-related and 117 (47.0%) were due to TRM. Sepsis was the most common cause of TRM and resulted in 75 deaths. The 5-year cumulative incidence of IRM was 2.4%, accounting for 75 (30%) of the 249 study deaths and 75 (64%) of the 117 TRM deaths. Sixty-eight percent of cases were associated with a bacterial infection (64% gram-negative), and 20% were associated with a fungal infection. The infectious pathogen was identified in 75% of the IRM cases (56/75), with dual pathogens identified in 3 cases. Gram-negative organisms represented the most common type of bacteria and accounted for 64% of bacterial infections. The most frequently detected specific bacteria were Pseudomonas (22%), *Escherichia coli* (20%) and Enterococcus. Fungal infections were the second most common cause of IRM (20%; 12 cases). The most frequently detected fungal pathogen was Aspergillus (8 cases), while in the remaining cases, the cause was mainly Candida (3 cases)^13^. Lehrnbecher et al. reported patients enrolled in the multicenter clinical trial AIEOP-BFM ALL2009 between 2010 and 2017. In a total of 6136 children (median age 5.2 years), 224 proven/probable IFDs (65 yeast infections and 159 mold infections) were reported. By logistic regression, the risk for proven/probable IFDs was significantly increased in children aged ≥ 12 years and those with a blast count ≥ 10% in the bone marrow on Day 15 (p < 0.0001 each). Children with proven/probable IFDs had 6-week and 12-week mortality rates of 10.7% and 11.2%, respectively. In the multivariate analysis, the hazard ratio for event-free and OS was significantly increased for children with proven/probable IFDs, those aged ≥ 12 years, and those with an insufficient response to therapy (p < 0.001 each). The authors recommend considering prophylaxis in some children with ALL who may be at increased risk of IFDs, including children with relapsed disease. This weak recommendation is based on the lack of baseline data on the incidence of IFDs in children with both newly diagnosed and relapsed ALL, as well as the lack of any randomized controlled trials in this patient population^14^. However, Groll et al. strongly recommend antifungal prophylaxis in patients with relapsed ALL and high-risk ALL based on randomized trials in adult populations^15^. In Poland, patients with ALL receive antifungal prophylaxis^16^.

In our study, we showed the profile of bacterial and fungal infections in pediatric patients with ALL in Poland in the pandemic period. These data indicate a high incidence of infectious complications in this population in the analyzed period. The pandemic probably contributed to the increase in bacterial infections. Based on our experiences and literature report, the directions of action should be to consider antibiotic prophylaxis, shorten the duration of hospitalization, and educate parents and medical staff about complications (mainly infections) during anticancer therapy. It is also necessary to continue clinical trials evaluating infection prophylaxis to improve outcomes in childhood ALL patients.

## Patients and methods

### Study group

This study was a retrospective analysis of 460 patients aged 1–18 years with newly diagnosed ALL who were treated in 17 pediatric hematology centers from January 2020 to December 2021 in Poland. In our previous paper, we included 1363 patients aged 1–18 years with newly diagnosed ALL who were treated in the same pediatric hematology centers between 2012 and 2017 in Poland. The study was approved by the Ethics Committee of Collegium Medicum in Bydgoszcz, Nicolaus Copernicus University in Torun, Poland. All methods were carried out in accordance with relevant guidelines and regulations. Informed consent was obtained from all subjects and/or their legal guardian(s).

### Treatment and supportive care

Pediatric patients received corticosteroids (during both the induction and postinduction phases) and multidrug chemotherapy in accordance with the treatment protocol used in Poland. Cytostatic drugs used in pediatric ALL treatment mainly include vincristine, cytarabine, anthracyclines, asparaginase and methotrexate. From 2012 to 2017, children with ALL were treated according to the ALL IC-BFM 2002 and ALL IC-BFM 2009 protocols, which were randomized trials of the IBFM-SG (International Berlin-Frankfurt-Munster Study Group). Both patients with B-ALL and T-ALL received prednisone during the induction phase and dexamethasone postinduction. The main difference between these protocols was that the new stratification based on minimal residual disease (MRD) evaluation was only applied in the ALL-IC BFM 2009 protocol. Antibacterial antibiotic prophylaxis was not used during the neutropenic phase, but Rh-G-CSF (recombinant human granulocyte colony-stimulating factor) was administered during sepsis in neutropenic or high-risk patients. All patients received oral cotrimoxazole (3 consecutive days/week) as prophylaxis for *Pneumocystis jirovecii* and antifungal prophylaxis (fluconazole)^10^.

In the present study, ALL patients were treated according to the AIEOP-BFM ALL 2017 protocol (International collaborative treatment protocol for children and adolescents with acute lymphoblastic leukemia). In this protocol, prednisone was applied during the induction phase, and dexamethasone was used postinduction for B-ALL patients. Initially, prednisone (7 days) was used during the induction phase, followed by dexamethasone for T-ALL patients with a good response to prednisone. Prednisone for the entire induction phase was administered for T-ALL patients with a poor response to prednisone. Dexamethasone was used postinduction for T-ALL patients. MRD was evaluated by flow cytometry (15 days after induction) and PCR (polymerase chain reaction) methods (33 days after induction and after 12 weeks of therapy). Supportive care for patients with bacterial infection and *Pneumocystis jirovecii* infection was similar to previous protocols. Antifungal prophylaxis was used in patients at risk for invasive fungal disease (IFD) development. Recommendations for antifungal prophylaxis in children based on the Polish Society of Pediatric Oncology and Hematology include posaconazole oral suspension (contraindicated when the patient is taking *Vinca alkaloids*), micafungin or fluconazole (active mainly against *Candida albicans*)^16^.

The studies only included patients with microbiological confirmation of the pathogen from infected sites. Fungal infection was classified as probable, proven and possible according to the European Organization for Research and Treatment of Cancer/Invasive Fungal Infections Cooperative Group (EORTC) and the National Institute of Allergy and Infectious Diseases Mycoses Study Group (MSG) criteria^17^.

### Statistical methods

The analysis was conducted in R software, version 4.0.5. (R Core Team (2021). R: Language and environment for statistical computing by R Foundation for Statistical Computing, Vienna, Austria) was used, assuming a significance level of α = 0.05. Patients were analyzed as two groups: patients with one infection and patients with more than one infection. Differences between groups were analyzed with the chi-square test, Fisher’s exact test or Mann‒Whitney’s U test, as appropriate. Differences between this study and the previous study were analyzed using the chi-square test. Kaplan‒Meier survival curves for the cumulative incidence of infections as well as OS were prepared, including a comparison between the analyzed groups based on chi-square log-rank test.

## Data Availability

All data generated or analyzed during this study are included in this article. Further enquiries can be directed to the corresponding author.
